# Review of Wireless RFID Strain Sensing Technology in Structural Health Monitoring

**DOI:** 10.3390/s23156925

**Published:** 2023-08-03

**Authors:** Gang Liu, Qi-Ang Wang, Guiyue Jiao, Pengyuan Dang, Guohao Nie, Zichen Liu, Junyu Sun

**Affiliations:** 1 School of Mechanics and Civil Engineering, China University of Mining and Technology, Xuzhou 221116, China; 2 State Key Laboratory for Geomechanics & Deep Underground Engineering, Xuzhou 221116, China

**Keywords:** SHM, RFID, strain sensing, wireless sensing, passive sensing

## Abstract

Strain-based condition evaluation has garnered as a crucial method for the structural health monitoring (SHM) of large-scale engineering structures. The use of traditional wired strain sensors becomes tedious and time-consuming due to their complex wiring operation, more workload, and instrumentation cost to collect sufficient data for condition state evaluation, especially for large-scale engineering structures. The advent of wireless and passive RFID technologies with high efficiency and inexpensive hardware equipment has brought a new era of next-generation intelligent strain monitoring systems for engineering structures. Thus, this study systematically summarizes the recent research progress of cutting-edge RFID strain sensing technologies. Firstly, this study introduces the importance of structural health monitoring and strain sensing. Then, RFID technology is demonstrated including RFID technology’s basic working principle and system component composition. Further, the design and application of various kinds of RFID strain sensors in SHM are presented including passive RFID strain sensing technology, active RFID strain sensing technology, semi-passive RFID strain sensing technology, Ultra High-frequency RFID strain sensing technology, chipless RFID strain sensing technology, and wireless strain sensing based on multi-sensory RFID system, etc., expounding their advantages, disadvantages, and application status. To the authors’ knowledge, the study initially provides a systematic comprehensive review of a suite of RFID strain sensing technology that has been developed in recent years within the context of structural health monitoring.

## 1. Introduction

Engineering structures may be damaged due to various natural and human factors during service life. Structural damage can be defined as the change of material and geometric properties of the whole structure or some structural component, e.g., the decay or decline of the structure in stiffness, boundary, strength, and connection conditions, which will affect the future performance for the whole structural system [1,2,3,4,5,6,7]. Sudden natural disasters such as earthquakes and hurricanes will cause extensive damage to engineering structures in a very short time. In addition, some human-made factors, such as terrorist bombings or other destructive behaviors, will also seriously influence the normal service function and structural safety. The aforementioned damage is a kind of sudden damage, and this kind of damage can lead to the loss of structural function in a short time, and even endanger structural safety. Additionally, in the long service life of the structure, because of material degradation, fatigue, steel corrosion, environmental changes, etc., there will be a certain degree of damage, which is cumulative damage, and, with the nature of slow accumulation, will also affect the structure safety and normal function.

In recent years, engineering structure collapse accidents occur frequently due to various natural and human factors. Structural health monitoring (SHM) plays a significant role in structural damage identification and reliability evaluation for in-service engineering structures. They can resort to sensor systems, data acquisition and processing systems, and computer software to monitor, diagnose and analyze the structural condition and safety performance of in-service engineering structures. Furthermore, a real-time health monitoring system for major engineering structures is established to timely identify structural damage and make corresponding early warning mechanisms, so as to give early warning to possible disasters.

In the context of structural health monitoring and structural state evaluation, the strain can reflect the local damage of the structure, and based on the strain monitoring data, the damage location, fatigue failure, and reliability evaluation of the structure can be carried out. Through the strain monitoring and state assessment of structures, strain and stress distribution can be obtained rapidly and effectively, and stress concentration area can be derived as soon as possible, which can help to realize the structural strength analysis, and evaluate the structure service condition to provide early diagnosis. The traditional strain monitoring techniques mainly include three methods: the resistance strain sensor method, the vibrating string strain sensor method, and the FBG optical sensor method. The resistance strain sensor is a resistance sensor with the resistance strain gauge as the conversion element. The elastic sensing element can be deformed caused by external excitation, and the attached resistance strain gauge will be deformed together. The resistance strain gauge can transfer the deformation into the resistance change so that a variety of physical quantities such as strain, acceleration, displacement, and temperature can be measured.

In the vibrating string strain sensor method, a certain length of adding a hyphen is tensioned between two installation blocks, and the installation block is fixed on the surface of the structure. When the structure is deformed, it causes a change in the steel string tension in the vibrating strain sensor, which leads to a change in the inherent collar of the steel. The electromagnetic wire solid excitation of the steel tension will produce food and reduce vibration because of the quota rate, and the structural strain can be obtained by reading only the changes of the steel tensioned inherent whisker. The fiber Bragg grating (FBG) sensor can change the light wavelength according to the change of strain and temperature. FBG exposes a small part of light-sensitive fiber under a light wave, and the fiber refractive index will change permanently according to the intensity of the light wave being irradiated.

However, in practical engineering applications, such traditional strain sensors have many weaknesses: the sensors need to be connected to each other by wired means and also the monitoring data are transmitted through wired methods. Thus, there needs more wiring, and the wiring operation is complicated and time-consuming. For instance, for the resistance strain sensor, the resistance change caused by strain is usually small, and the transmission of weak resistance changes requires a high-quality wire, so the wire cost is greatly increased. At the same time, too-long wires are susceptible to noise when they transmit weak resistance changes, which greatly reduces the accuracy of the signal. In addition, data acquisition equipment for traditional strain sensors is generally expensive. Therefore, in civil engineering applications, with the aim to overcome the shortcomings of the traditional strain sensors, strain sensing technology based on radio frequency identification (RFID) came into being. This technology can not only make up for the shortcomings of traditional monitoring methods such as complex wiring and high cost, but also overcome the shortcomings of other wireless sensor systems such as cumbersome, low efficiency, and large energy consumption, which can realize the low-cost, passive, and wireless strain measurement. This study reviews a suite of the fundamental literature to demonstrate the recent development of RFID strain sensing technologies. The RFID technology is first introduced in detail, and the basic principle of the RFID technique for wireless strain sensing is also described. Further, the design and application of various kinds of RFID strain sensors in SHM are presented, expounding their advantages, disadvantages and application status.

The organization of this article is as follows. The research background is presented in Section 1. Section 2 outlines the RFID technology, including the introduction of the RFID electronic tag and reader. In Section 3, the design and application of RFID strain sensors in SHM are presented. In the end, the conclusion and discussion of RFID strain sensing technology are drawn in Section 4.

## 2. Overview of RFID Technology

### 2.1. RFID Technology

An RFID system is generally composed of an RFID electronic tag, an RFID reader, and an RFID tag antenna [8,9,10]. RFID electronic tag is generally a low-power integrated circuit with coil, antenna, and memory and control system. An RFID reader, also known as a signal receiver, is a device employed to read/write RFID tag information. An RFID reader is composed of several parts: a processor, an operation system, a memory, and a transceiver module. An RFID tag antenna serves as the transponder antenna of the RFID electronic tag, which can transmit RF signals between the RFID electronic tag and the RFID reader. An RFID tag antenna can be classified into metal etching antenna, printing antenna, copper-plated antenna, etc. In recent years, with the development of nanotechnology, a nano silver paste antenna has appeared.

Regarding the working principle of RFID, RFID belongs to one of the wireless communication technology, utilizing radio frequency signals to achieve the transmission of information and the acquisition of relevant data wirelessly. Specifically, the RFID reader is employed to identify the radio frequency signals, and the RFID tag is carried with the encoded information and responds through the built-in antenna. The RFID reader will transmit radio frequency signals of a specific frequency. When the RFID tag entering the identification range receives the radio frequency signals, the RFID tag is endowed with energy to activate the work. The RFID reader will collect radio frequency signals from the RFID tag by the receiving antenna and sends the signals to the host equipment after the demodulation processing. The tag number and other information can be identified by the host system, which will execute the corresponding instructions according to different settings.

### 2.2. RFID Electronic Tag

As a non-contact identification and communication technology, an RFID electronic tag can identify the target and acquires data through RF signals automatically [11]. The encoding of RFID electronic labels is stored in read-only or read/write formats on integrated circuits. In particular, the read/write modes of RFID electronic tags are achieved by wireless electronic transmission [12].

The RFID electronic tag generally comprised a tag antenna and coupling components [13]. According to different working frequencies, RFID tags are generally classified into the super-high-frequency RFID tag (2.45 GHz, 5.8 GHz), the ultra-high-frequency RFID tag (300 MHz~1000 GHz), the high-frequency RFID tag (3~30 MHz), and the low-frequency RFID tag (30~300 KHz). Based on energy supply modes, RFID electronic tags can be classified into the active RFID tag, the passive RFID tag, as well as the semi-active RFID tag. The active electronic tag needs to be powered by batteries to maintain the required work, and it generally has a longer reading distance. However, the disadvantages of the active electronic tag are the need for a power supply and the limited battery life. The passive electronic tag, which is battery-free, relies on microwave signals received from an RFID reader. Compared with the active electronic tag, the passive electronic tag has limited reading distance and application scenarios. The semi-active electronic tag is equipped with a battery, but the battery only supplies power to the internal circuit of the tag. Under normal circumstances, the semi-active electronic tag often lies dormant and does not send out RFID signals. When entering the activation range, the semi-active RFID tag is activated and begins to work [14]. There are various sizes and working frequencies for RFID electronic tags, which can be attached to the surfaces of the target object and store the relevant data and information. Each RFID tag owns a corresponding tag number, and RFID tags can be attached to the target object for object identification. In addition, the RFID tag can store the relevant data and information and then transmit this information to the RFID reader. RFID electronic tag is generally a low-power integrated circuit with a coil, a tag antenna, and a memory and control system [15]. As the transponder antenna, the tag antenna can transmit RF signals and data between the tag and the reader [16].

### 2.3. RFID Reader

RFID readers can identify RFID electronic tags and can realize wireless communication technology. As RFID technology matures and is widely used, the demand for RFID readers is also increasing [17]. Currently, the stability and reliability of the RFID reader application still need to be improved. Therefore, how to design a stable performance that is cost-effective and adapts to the market demand of RFID readers is a problem worth discussing [18].

RFID readers can realize the RFID tag identification, as well as the read or write operation for monitoring data. The RFID reader plays an important role in the RFID system. Firstly, the working frequency of the whole RFID system is determined by the frequency of RFID readers. Secondly, the transponder is passive and its working energy is extracted from the RF signal sent out from RFID readers, so the RFID reader power directly affects the distance of the radio frequency identification [19].

The RFID reader generally comprises an RFID transmitter and receiver, an RFID reader antenna, and a control system. The control system is mainly realized by the microprocessor [20]. The communication protocol of the reader and transponder, cyclic redundancy check, data encryption, and coding and decoding can all be realized by the control system software [21]. The functions of the RFID reader are as follows: identifying RFID tag numbers of the transponder, executing the input command, controlling the communication with the transponder, signal coding and decoding, controlling the display interface, implementing anti-collision algorithm, and two-way identity authentication between the transponder and reader [22]. The main function of the RFID transmitter and receiver is generating RF signals to activate and power the transponder, modulation of the transmitted signal for transmitting data onto the transponder, and receiving and demodulating the RF signal returned from the transponder [23]. The communication frequency of RFID readers is generally fixed and only meets one or two ISO protocol standards. The same frequency and the same communication protocol are the necessary conditions for normal communication between tags and readers. For labels that work in a certain frequency band and conform to a certain communication protocol, only readers that meet this frequency and communication protocol can be selected to read RFID tags [24].

## 3. Design and Application of RFID Strain Sensors in SHM

### 3.1. Passive RFID Strain Sensing Technology

The strain sensor based on passive RFID technology is a relatively common type, with the advantage that it can realize completely infinite passive strain sensing without an external power supply. However, the transmission distance is relatively short. Dey investigated a chipless passive RFID strain sensor tag with a traditional planar antenna, which can be used in the SHM applications areas such as soil strain monitoring, where the object or structure is exposed to less external traction force. Khan et al. [25] introduced a prototype of a low-energy high-temperature exposure sensor. It is a temperature-sensitive passive UHF RFID tag, which will bend forward if the temperature rises. For the initial design, the sensor tag has an interrogation range of more than 6 m. After bending, the reading range is significantly reduced (about 2–3 m) due to the change in the backscattered power of the sensor tab.

**Figure 1:**
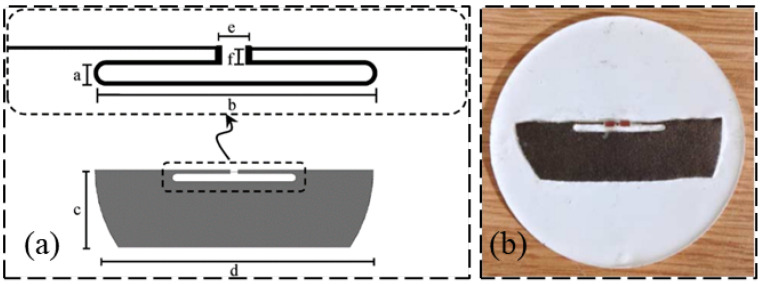
(**a**) Geometry of the designed RFID strain sensor; (**b**) designed RFID tag [27].

Carbon nanotubes have good performance and the use of this material can make the sensor detection range larger and the effect better. Benchirouf et al. [26] proposed a wireless strain sensor made of carbon nanotube dispersion on an inkjet printing flexible substrate. A new method based on a multi-wall carbon nanotube layer printed coil is used to draw plane coil patterns on a flexible substrate to realize wireless strain measurement. Rakibet et al. [27] designed a new passive RFID tag structure for strain sensing, and Figure 1 shows the geometry of the proposed RFID strain sensor and tag. In this figure, “a” is the slot width, “b” is the slot length, “c” is the tag width, “d” is the tag length, “e” is the chip length, and “f” is the feed line thickness.There are two strain gauge-based configurations. The first one employs a flexible substrate, which can record the bending degree response. The second one employs a specially designed structure that can measure absolute forces.

**Figure 2:**
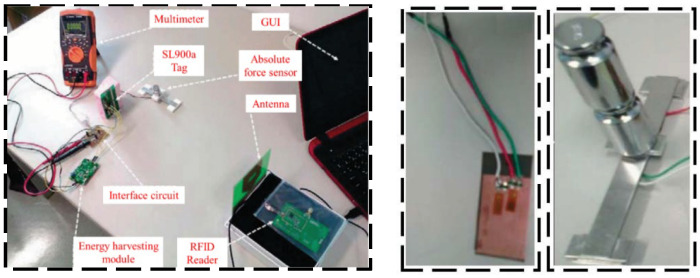
Measurement for absolute force or bending level [28].

**Figure 3:**
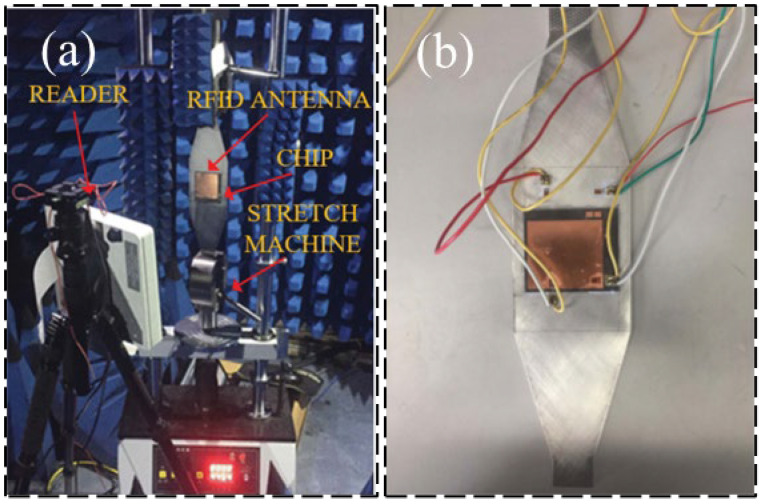
(**a**) Laboratory measurement setup; (**b**) transmission efficiencies test through a strain gauge [29].

**Figure 4:**
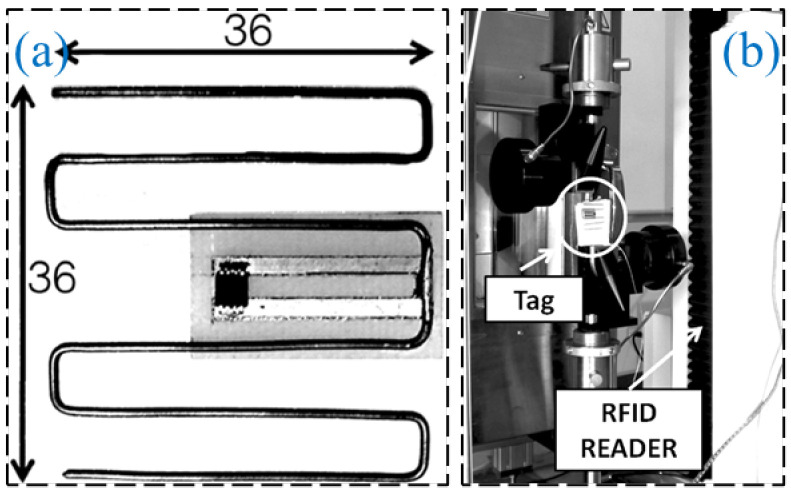
(**a**) Prototype of RFID tag; (**b**) RFID strain sensor laboratory test [30].

With the aim to verify the measurement reliability of passive RFID sensors, Ahmed et al. [28] proposed a novel RFID sensor tag combined with strain gauges, and two strain gauges were designed and tested with the capacity to monitor respectively bending and absolute force, as shown in Figure 2. The proposed design can eliminate the temperature effect and can adjust the sensitivity of the measurement. The experiment results show that the two sensors both have good response linearity and measurement reliability is high. He et al. [29] proposed a new rectangular RFID patch antenna for structural strain monitoring wirelessly and passively (shown in Figure 3). When designing the RFID antenna, a microstrip antenna with a row of short-circuit holes was used which can not only reduce the antenna size but also improve the frequency shift sensitivity. Occhiuzzi et al. [30] proposed a passive UHF RFID sensor for strain sensing. As shown in Figure 4, this kind of equipment has low cost and sub-millimeter resolution, which can be used in the SHM field for engineering structures during extreme events.

**Figure 5:**
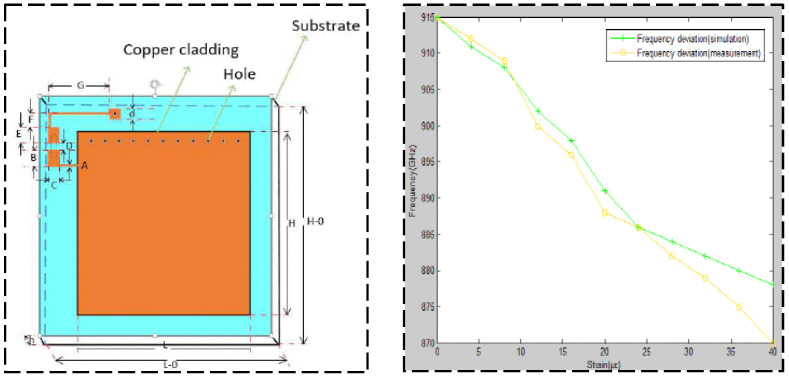
Structure of strain antenna and simulation results [32].

**Figure 6:**
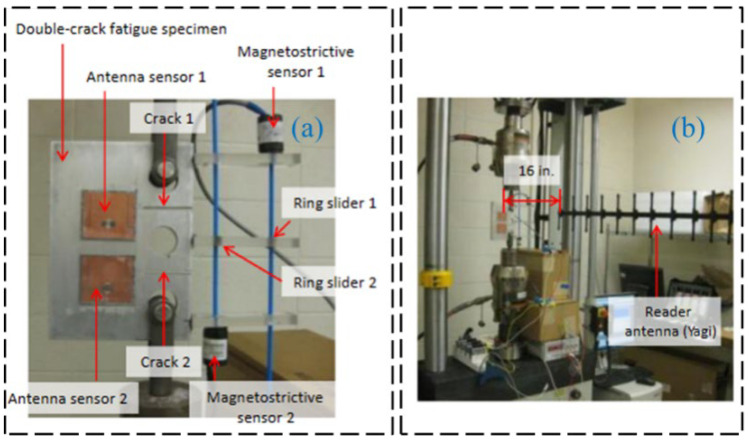
Experimental setup for the fatigue test of the proposed passive RFID antenna: (**a**) experimental specimen; (**b**) laboratory setup [33].

**Figure 7:**
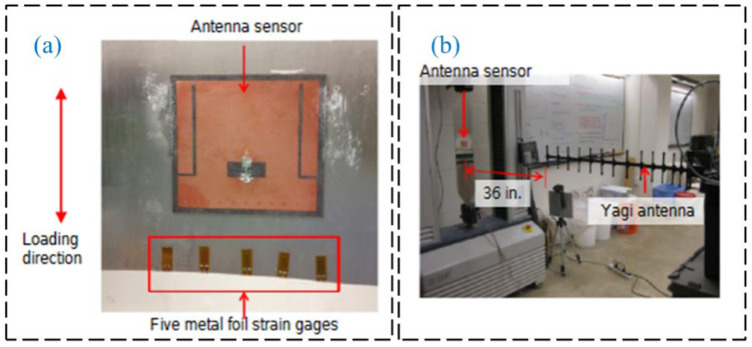
(**a**) RFID antenna sensor attached on a specimen; (**b**) laboratory test scenario [33].

Regarding passive RFID technology, many studies investigated communication and data storage algorithms for efficient wireless data collection. Also, much research focused on improving transmission distances for better engineering applications. Gonek et al. [31] proposed a wireless sensing platform using RFID technology as a fully passive and wireless communication interface between any connecting sensors and RFID readers. By improving the communication and data storage algorithms and testing various kinds of RFID readers, the effective data rate of 18 kbit/s is realized. Wan et al. [32] proposed a wireless passive UHF rectangular patch antenna with good linear polarization, which achieved an ideal gain and allowed a larger measurement distance. Its application in SHM can provide great convenience for field operations. The structure of the strain antenna and simulation results are shown in Figure 5. As shown in Figure 6 and Figure 7, Yi et al. [33] designed a completely battery-free (passive) and wireless slotted patch antenna sensor and tested its performance in the laboratory. The sensor operation is passive without the need for a battery and wireless because the operation energy comes from a wireless reader interrogation signal.

### 3.2. Active RFID Strain Sensing Technology

**Figure 8:**
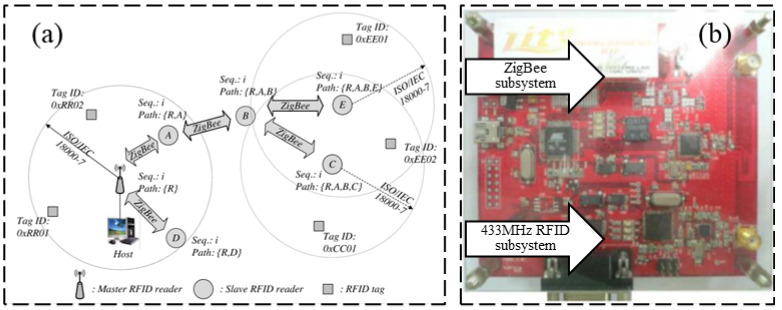
(**a**) Multihop deployment scheme for active RFID readers; (**b**) reader collision avoidance design [34].

Compared with passive RFID sensors, the active RFID strain sensor has a higher working frequency, generally equipped with external power. Correspondingly, active RFID sensors also consume more energy. The advantage of this active RFID strain sensing technology is the long transmission distance, which is especially suitable for practical engineering applications. Cho and Baek [34] proposed an RFID reader collision avoidance strategy for multihop deployment of active RFID readers, as shown in Figure 8. The proposed strategy can avoid multiple reads for an RFID tag, which can extend the serving life for RFID tags.

**Figure 9:**
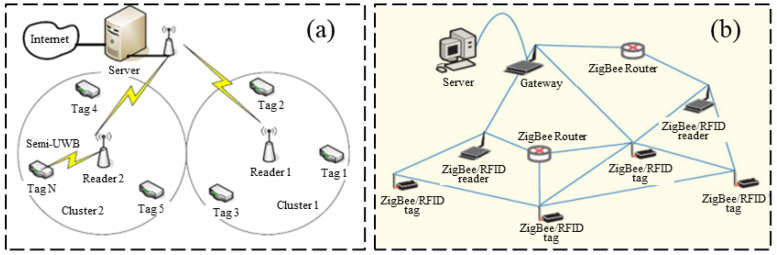
(**a**) Generic sensor networks architecture; (**b**) active RFID devices with the incorporation of ZigBee [35].

In the context of sensing technology, wireless sensor networks have the advantages of low energy consumption, inexpensive hardware, and small size of wireless multifunctional sensor devices. Figure 9 shows a wireless sensor network that uses active RFID technology to transmit data between the RFID tag and reader, which then forwards the data to the WLAN through special features with a transmission distance of about 10 m [35].

**Figure 10:**
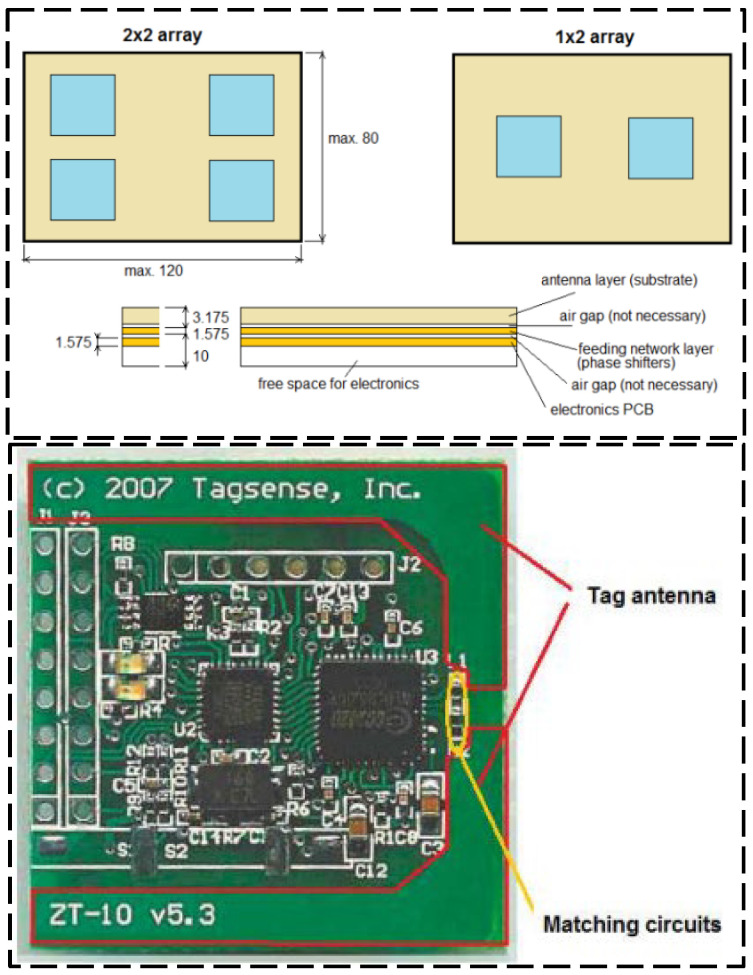
Designed a simple RFID tag and detailed view of the RFID tag [36].

There is a significant interrogation range for the active UHF RFID tags, which directly obtain the energy from the radiated radio frequency power. The design of the RFID tag antenna has a critical importance on the final interrogation range [36]. Active RFID tags are set up to increase communication range, so the radio frequency power should also rise. Active UHF RFID tags containing antennas with adaptive beam deflection must be of sufficient size, circuit complexity, and long operation time. Figure 10 shows the design concept for an active UHF RFID tag.

### 3.3. Semi-Passive RFID Strain Sensing Technology

**Figure 11:**
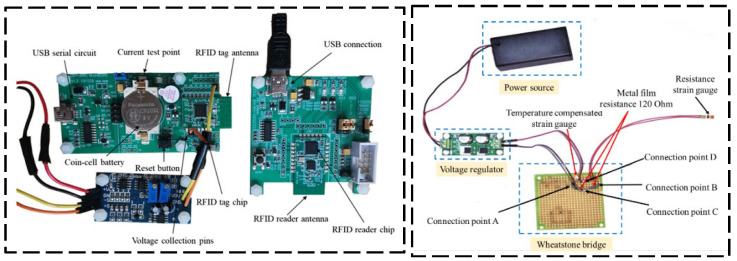
Designed RFID strain sensors with a temperature compensation gauge [37].

**Figure 12:**
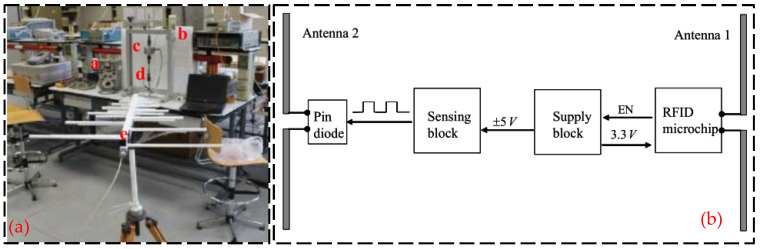
(**a**) RFID experimental test setup: a—manual compensator; b—steel frame; c—the load cell; d—the instrumented specimen; e—the log-periodic antenna. (**b**) scheme of the RFID sensor tag [38].

Semi-passive RFID strain sensing technology incorporates the advantages of both active and passive RFID tags. The semi-passive RFID tags are in a dormant state for most cases with extremely low power consumption. Only when the tag enters the activation signal range, the semi-passive RFID tag is activated, and the tag will start to work. The advantages of this technology are longer transmission distance, low power consumption, and longer service life, but the disadvantage is that the design is more complex. Wang et al. [37] proposed a new semi-active RFID strain sensor with dual interrogation mode to realize wireless strain sensing for longer-distance query transmission. As shown in Figure 11, the proposed design scheme includes a wireless strain transfer module and strain monitoring module for an improved Wheatstone bridge. The designed RFID strain sensor has temperature self-compensation characteristics and can provide the advantage of the long interrogation distance, which is especially suitable for the SHM application of practical engineering structures. In order to overcome the limitations and shortcomings of sensors based on wireless sensor networks, as shown in Figure 12, DiGiampaolo et al. [38] proposed a semi-passive wireless strain gauge sensor. It can carry out long-distance measurements and has the ability to deal with fast time-varying phenomena, e.g., vibration responses.

**Figure 13:**
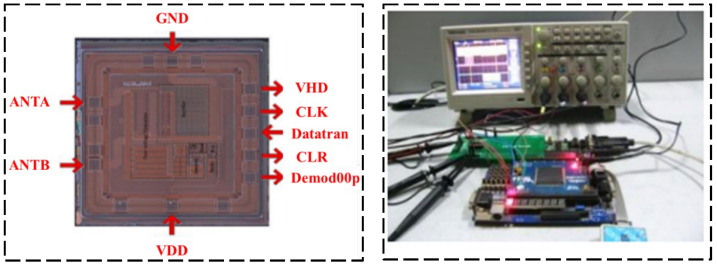
Die photo of the test platform [39].

**Figure 14:**
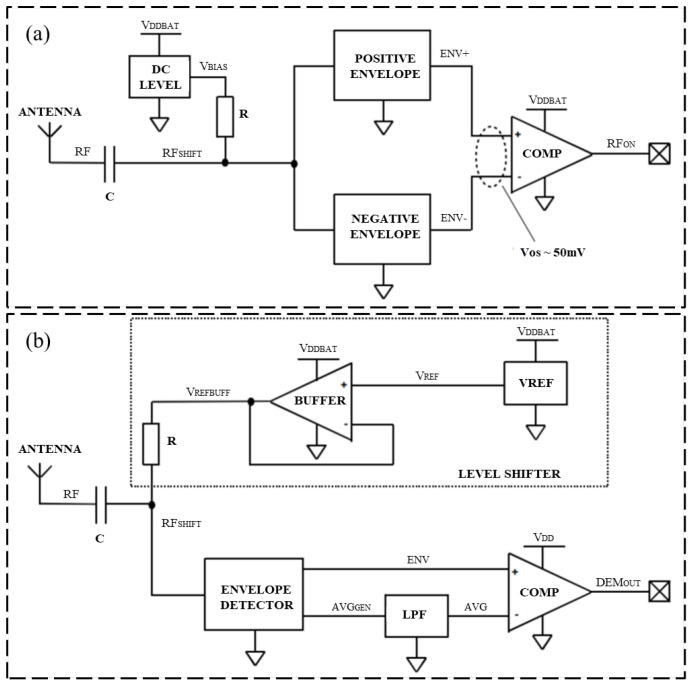
(**a**) Designed radio frequency detector circuit; (**b**) designed demodulator circuit [40].

**Figure 15:**
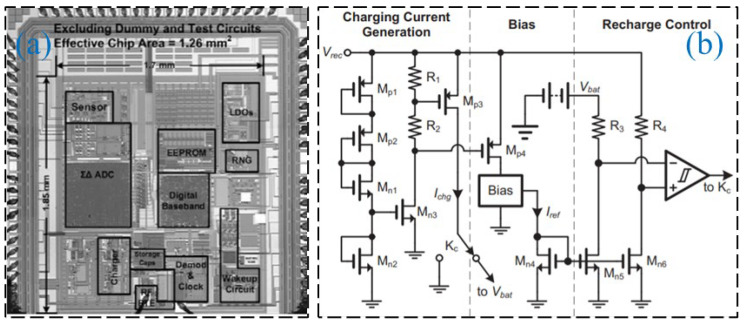
(**a**) Chip micrograph; (**b**) charger schematic [41].

Regarding the semi-active RFID tag circuit design, Zou and his research team [39] presented a novel analog front-end circuit for a semi-passive high-frequency RFID tag, which can work normally in the magnetic field with an intensity of 0.3 A/m. Experiments show that the proposed RFID design can meet the demands of a high-frequency semi-passive RFID tag, which made it possible to implant the sensor in the semi-passive RFID tag. The laboratory test platform is shown in Figure 13. Henes Neto [40] proposed a semi-passive UHF RFID sensor tag. This semi-passive RFID tag was manufactured using 0.18 μm standard CMOS technology, which implements a low-power PMU and has a high RF power sensitivity. The proposed RF detector and demodulator circuits are shown in Figure 14. Che et al. [41] designed a semi-passive ultra-high frequency RFID tag circuit with an on-chip sensor, which implements new power management techniques to increase battery service life. The chip micrograph of the tag and the schematic of the charger is shown in Figure 15.

### 3.4. Ultra High-Frequency RFID Strain Sensing Technology

**Figure 16:**
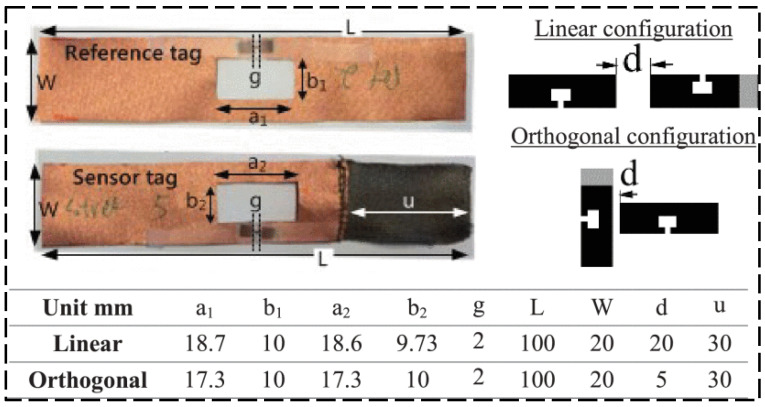
Samples of manufactured tags and studied sensor configurations [43].

**Figure 17:**
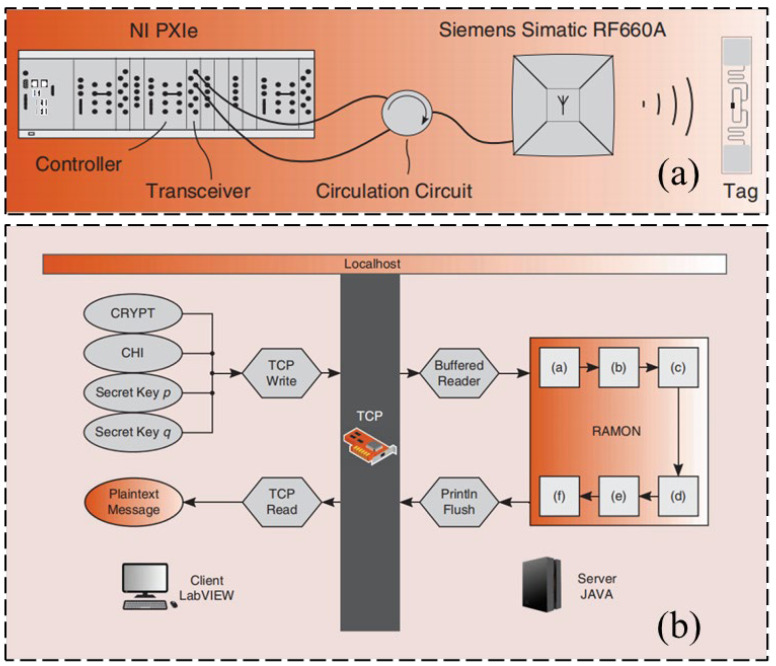
(**a**) UHF RFID hardware setup; (**b**) visualized software procedure [44].

UHF RFID techniques have the advantage of long transmission distance, but the overall sensing system has high energy consumption. This section will present some examples to show the achievements made in the field of UHF RFID strain sensing technology. Yang et al. [42] proposed a passive UHF RFID sensor tag to detect solutions with a conductivity of smaller than 0.5 S/m. As the conductivity is smaller than 0.5 S/m, the performance of the RFID tag decreases rapidly. Chen et al. [43] proposed a new semi-passive UHF RFID wireless sensor for strain measurement, including a wireless technology based on a traditional strain gauge and Wheatstone bridge. The samples of manufactured tags and studied sensor configurations are shown in Figure 16. Compared with a wired strain sensor, it has better response linearity, sensitivity, and accuracy, and can increase sensitivity according to user requirements. Saxl and his team [44] worked on the integration of sensors in UHF RFID tags for a variety of different applications. The proposed UHF RFID reader details are shown in Figure 17a and one of the application’s visualized procedures is shown in Figure 17b. Research on determining the location of RFID tags is also carried out.

**Figure 18:**
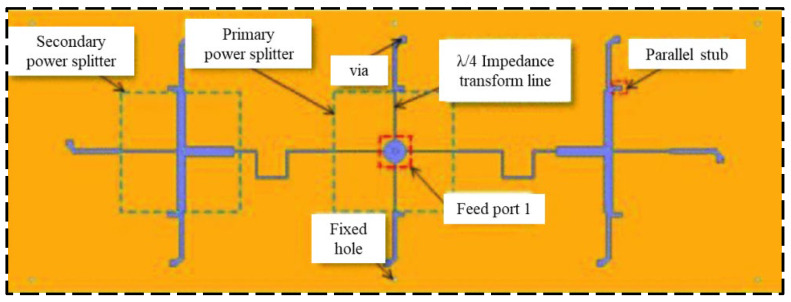
Bottom view of the configuration of the designed antenna [45].

**Figure 19:**
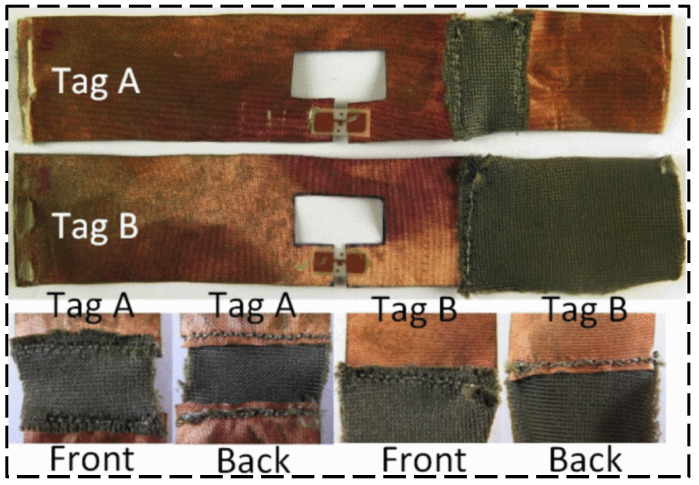
Tested passive RFID tags based on electrospinning antenna [46].

**Figure 20:**
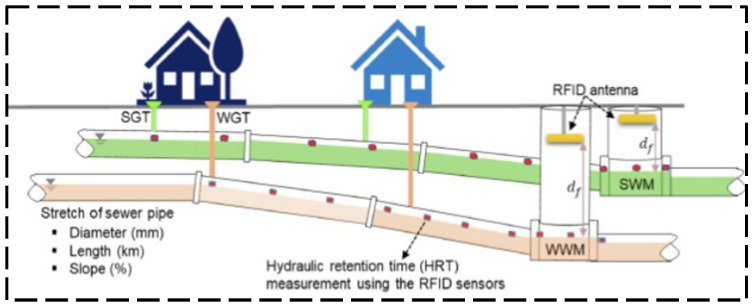
Field experiments for the proposed RFID systems [47].

**Figure 21:**
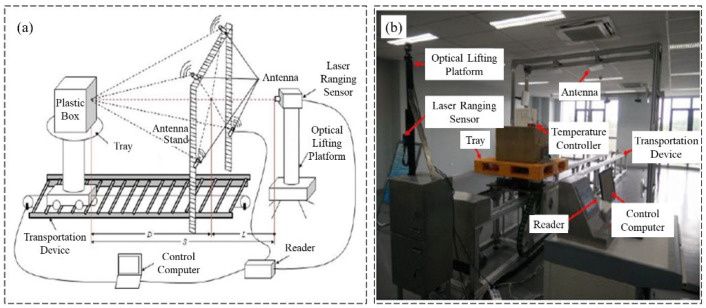
(**a**) Proposed UHF RFID system; (**b**) experiment setup [48].

A near-field UHF RFID reader antenna was designed by Qian et al. [45] with a long interrogation distance. The proposed antenna structure is shown in Figure 18, which exhibits a uniform and stable magnetic field distribution for a large interrogation range. As shown in Figure 19, Long et al. [46] designed a passive UHF RFID strain sensor tag. The antenna body is made of non-stretchable and highly conductive materials, and the stretchable conductive fabric is obtained by stitching the two fabrics with conductive wires. A flushable sensor [47] based on UHF RFID for rapid surveys of sanitation and stormwater pipelines is shown in Figure 20 for intelligent monitoring applications in sewage systems. In order to investigate the influence of temperature on UHF RFID system dynamic reading performance, an RFID detection system with temperature control was designed by Yu et al. [48] (shown in Figure 21), and the influence of temperature on reading performance was studied.

### 3.5. Chipless RFID Strain Sensing Technology

**Figure 22:**
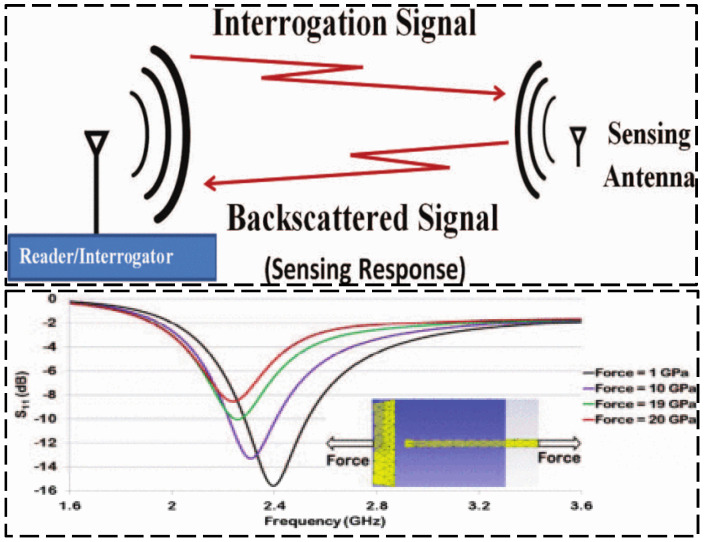
Interrogation system for the chipless RFID strain sensing antenna and sensing response diagram for the designed RFID antenna [53].

**Figure 23:**
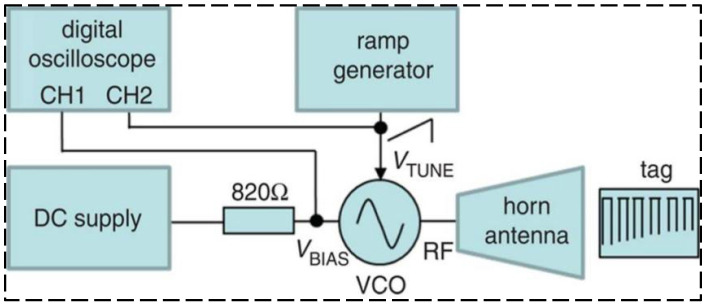
Single voltage-controlled oscillator chipless RFID reader [54].

**Figure 24:**
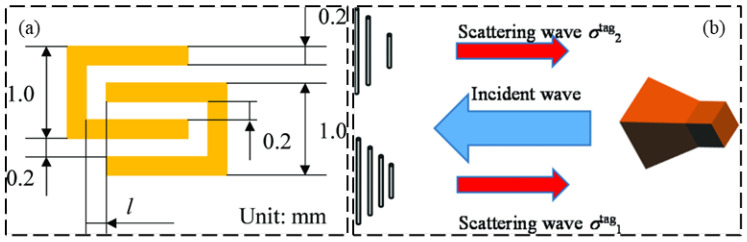
(**a**) Proposed chipless RFID strain sensing tag; (**b**) chipless RFID interrogation technique [55].

While chipped RFID technology is well developed, current research is focused on finding solutions providing a superiority with respect to lower cost, a coping strategy is to eliminate the use of RFID chips in the RFID tag design, generating a device which is named “chipless RFID” [49,50]. Chipless RFID technology has sensing capabilities while remaining low-cost, printable, and high mass production efficiency [51,52]. As shown in Figure 22, Shuvashis [53] studies the design of a chipless RFID strain sensor with a traditional planar antenna. The maximum strain of the antenna is determined by the structural deformation under various types of strain response, and the acceptability of the design is obtained, which is more adaptable to energy harvesting and more compatible with harsh SHM environments. A single voltage-controlled oscillator chipless RFID reader is provided in Figure 23, which can detect the 10-bit RFID tag with a working frequency range of 2.4–3.4 GHz. Compared with the more sophisticated vector network analyzer arrangement, The proposed single voltage-controlled oscillator solution generates a significant simplification for the chipless RFID reader design [54]. As shown in Figure 24, Watanabe [55] introduces a strain sensor based on a millimeter wave band chip-free RFID tag. The proposed RFID tag frequency is influenced by the change in the tag shape. The RFID strain sensor tag shape is optimized by simulation and analysis, and the ideal resonant frequency can be obtained.

### 3.6. Wireless Strain Sensing Based on Multi-Sensory RFID System

**Figure 25:**
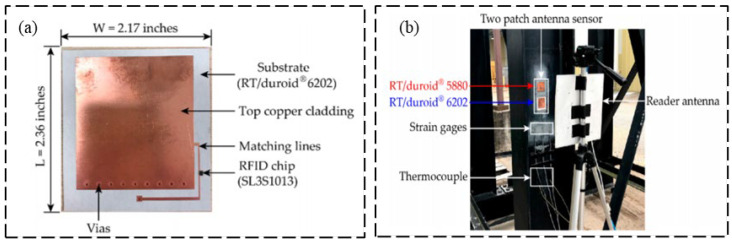
(**a**) Designed passive RFID strain sensing antenna; (**b**) experimental test [56].

With the improvement of RFID sensor technology, single sensing function has been difficult to meet the engineering requirements. Therefore, engineers are exploring the integration of multiple physical quantity monitoring in one RFID tag to form a multi-sensory RFID system. This multi-sensory RFID system shares one RFID tag, which can reduce the cost of the sensor, in addition, it can measure multiple physical quantities at the same time, and the integration is higher. However, this kind of sensor design is more complex and difficult to design. This section will introduce the RFID-based multi-sensor system which is able to simultaneously record strain and other structural responses. As shown in Figure 25, Li and Wang [56] designed an RFID antenna to improve strain and crack sensing performance by releasing the effect of temperature fluctuation.

**Figure 26:**
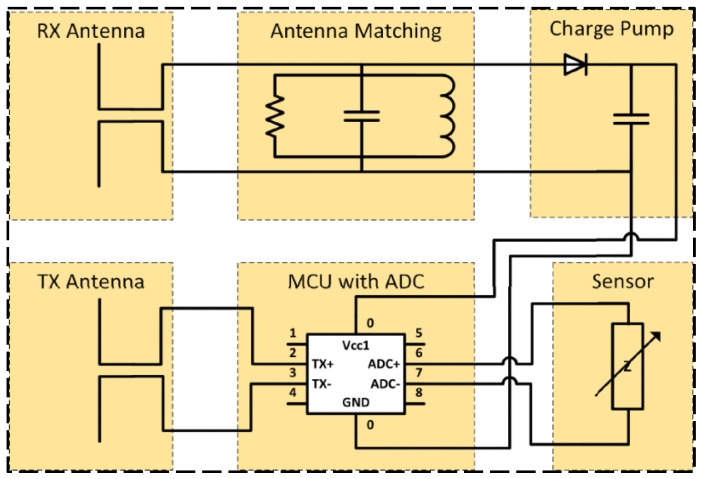
Self-powered RFID sensor through energy harvesting technique [57].

**Figure 27:**
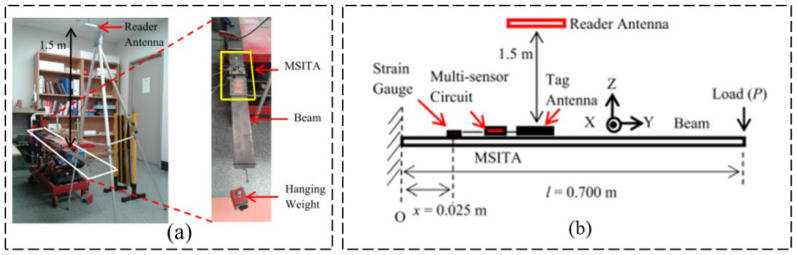
RFID multi-sensory system recording vibration and strain responses: (**a**) laboratory measurement; (**b**) experiment schematic diagram [59].

**Figure 28:**
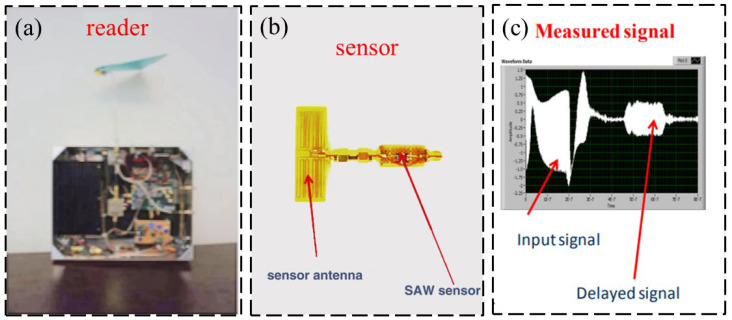
(**a**) Employed RFID reader; (**b**) fabricated SAW-RFID sensor system; (**c**) recorded signals [60].

For most multi-sensory RFID systems, two or more types of structural responses are generally usually monitored simultaneously (e.g., monitoring of strain and acceleration responses). For other cases, structural responses and environmental parameters may be monitored simultaneously (e.g., monitoring of strain and temperature). As shown in Figure 26, Cook et al. [57] introduced the RFID sensor technology based on chip and non-chip radio frequency identification principle and introduced various implementations of radio frequency identification sensors based on radio frequency identification technology, which can be employed to measure strain, water quality, and temperature simultaneously. Chakrabartty et al. [58] introduced the second generation of RFID-compliant, hybrid electricity structure health management sensors which can simultaneously monitor the structural responses and ambient parameters, which experience zero downtime in SHM mechanical events of interest. Jayawardana et al. [59] developed a wireless RFID-enabled multi-sensor infrastructure health monitoring system for the measurement of dynamic acceleration and strain simultaneously, and the proposed sensor system is shown in Figure 27. As shown in Figure 28, Li et al. [60] designed a wireless surface acoustic RFID sensor system for strain and temperature measurement with passive, wireless, and high-temperature resistance characteristics.

### 3.7. Wireless Strain Sensing Based on Other RFID Technologies

**Figure 29:**
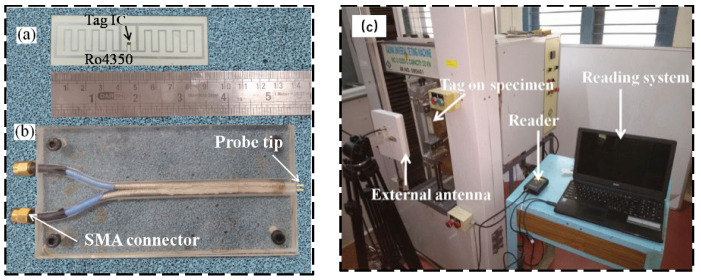
(**a**) Designed RFID sensor tag. (**b**) Text fixture for fabricated RFID tag characterization. (**c**) Experimental setup with the tensile specimen [61].

In addition to the aforementioned RFID strain sensing technologies, many researchers have explored other different types of RFID strain sensing technologies, including wireless strain sensing based on reusable RFID technology, metallic environments RFID technology, virtual RFID technology, etc. Regarding the wireless strain sensing based on reusable RFID technology, as shown in Figure 29, Chakaravarthi et al. [61] designed an RFID sensor antenna for structural health monitoring, and there are two resonant frequencies for the proposed antenna. The first one is sensitive to the variable force direction, which can be employed to monitor the strain direction. The second one is contrary to the first one in sensitivities, which can be employed for the structural strain amplitude measurement. The developed RFID sensors can be used for wireless strain measurement and SHM of metallic components and structures.

**Figure 30:**
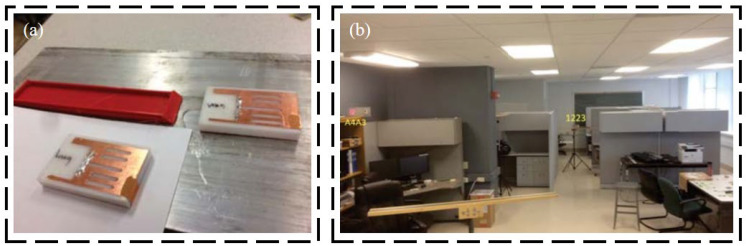
(**a**) Double slit antenna tags; (**b**) experimental setup scenario [62].

In metallic environments, the RFID tag performance will degrade. In order to enhance the RFID sensor performance in harsh metallic environments, an RFID tag antenna design with a ground plane can be adopted. Thus, Zawodniok et al. [62] used double-slit antennas for the RFID tag design with a ground plane (shown in Figure 30), and two RFID reader antennas were used to construct a phased array to increase the overall RFID interrogation range. The experimental results demonstrated that a 30 feet interrogation range can be achieved.

**Figure 31:**
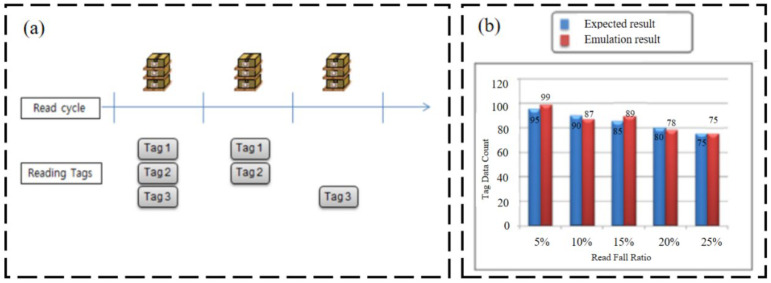
(**a**) Example of read failures; (**b**) read fail ratio [63].

Regarding wireless strain sensing based on virtual RFID technology, Lee et al. [63] proposed a virtual RFID reader mechanism, and this mechanism can emulate a physical RFID reader with the consideration of communicational characteristics between the RFID reader and tags (shown in Figure 31).

In order to introduce the reviewed RFID sensors in more detail, the following table has been summarized to describe the test performance, technical data, and features of every RFID solution, as well as the maturity level. It can be concluded from Table 1 that most RFID sensors are investigated and tested in the laboratory stage for SHM applications, and relatively few studies have been applied to the SHM of on-site practical engineering structures. Actually, only when the RFID tag is within the range of RFID reader recognition, the RFID reader can activate the RFID sensor tag through electromagnetic waves, and the RFID sensor enters the working mode to carry out strain data collection and wireless communication for SHM application. When the RFID reader is turned off or outside the identification distance, the RFID tag enters sleep mode and no longer performs strain sensing, so RFID strain sensors are generally used for discontinuous strain measurements in SHM applications compared to traditional wired strain sensors. In addition, the wireless transmission distance of current RFID strain sensors is relatively short. Therefore, for continuous and long-distance SHM, RFID wireless strain sensing technology still has its limitations in practical real-world engineering applications. Also, the RFID reading range, reliability, and measurement accuracy for SHM applications need to be further investigated to improve the field performance for on-site practical engineering.

The influencing factors that restrict the application of RFID sensors in the field of structural health monitoring mainly include the following aspects: the wireless transmission distance of RFID sensors is relatively short; battery life technology restricts the development of active RFID sensors; and, in terms of electromagnetic interference, there are many influencing factors affecting electromagnetic wave in the real environment, so it is hard to extract stable and accurate electromagnetic information for sensing and detection. The sampling frequency and test accuracy of the RFID sensing system still need to be improved for SHM field applications.

## 4. Conclusions

This study reviews a suite of the fundamental literature to demonstrate the recent development of RFID strain sensing technologies. The RFID technology is first introduced in detail, and the basic principle of the RFID technique for wireless strain sensing is also described. Further, the design and application of different kinds of RFID strain sensors in SHM are presented including passive RFID strain sensing technology, active RFID strain sensing technology, semi-passive RFID strain sensing technology, Ultra-High-Frequency RFID strain sensing technology, chipless RFID strain sensing technology, and wireless strain sensing based on multi-sensory RFID system, etc. Compared with traditional monitoring technologies, the RFID strain sensor avoids large-scale tedious wiring, and also the operation process is simple and the monitoring efficiency can be improved. With the comprehensive and thorough review, it can be concluded that there exists a prominent trend in employing wireless RFID strain sensing technology for monitoring, damage detection, and structural control. Although these RFID strain sensors have begun to generate wireless and passive strain monitoring for engineering structures, there still exists a high need for investigating the reliability and robustness of the RFID sensing technology with regard to real-time and online monitoring of the large engineering structures. Generally, the continuous long-term data acquisition capability of RFID sensors is insufficient compared to traditional wired strain sensors. In addition, the wireless transmission distance of current RFID strain sensors cannot be too far. For completely passive RFID strain sensors, its wireless transmission distance is small, generally within a range of a few or a dozen meters, which limits its application in practical engineering. For semi-passive or active RFID strain sensors, the wireless transmission distance can reach a range of 100 m. Therefore, for continuous and long-distance strain monitoring, RFID wireless strain sensing technology still has its limitations in practical real-world engineering applications. The use of chipless RFID sensing technology, and multi-sensory RFID system to harness the utility of low-cost and highly efficient sensors for strain monitoring is still in the nascent stage. In the field of chipless RFID strain sensing research, emerging smart materials (e.g., graphene, carbon nanotubes, etc.) need to be developed to improve the gain of RFID tag antennas and improve electromagnetic characteristics. In addition, the RFID reading range and the 3D printing technology of RFID tags need to be further investigated to improve the field performance for on-site practical engineering.

## Figures and Tables

**Table 1:** Technical data and features of the reviewed RFID sensors.

No.	Technical Data and Features	Maturity Level
[25]	Low-energy high-temperature exposure sensor with a read range of more than 6 m. After bending, there is a significant decrease in the read range (to around 2–3 m). The backscattered power changes from −36 dBm to −43 dBm.	Laboratory
[26]	Wireless passive RFID strain sensor with carbon nanotubes smart materials and inkjet technology.	Laboratory
[27]	Passive wireless RFID strain sensor with a barium titanate-loaded polydimethylsiloxane substrate.	Laboratory
[28]	Passive UHF RFID sensor with flexible substrate.	Laboratory
[29]	Novel rectangular patch antenna in a wireless and passive manner.	Laboratory
[30]	Completely passive UHF RFID sensor with low-cost and sub-millimeter resolution.	Laboratory
[31]	Completely passive wireless communication interface with an effective communication and data storage rate on the level of 18 Kbit/s.	Laboratory
[32]	Wireless and passive UHF rectangular patch antenna.	Laboratory
[33]	Completely battery-free (passive) and wireless sensor with a slotted patch antenna.	Laboratory
[34]	Reader collision avoidance for multihop deployment of active RFID system which complies with ISO/IEC 18000-7.	Laboratory
[35]	Review study on RFID wireless sensors.	NA
[36]	An energy-efficient RFID tag and the antenna system, which use the technology of adaptive beam forming.	Laboratory
[37]	Dual-interrogation-mode RFID strain sensor with a reading range of 80 m, which can automatically switch between passive modes with low power consumption and active UHF modes.	Laboratory
[38]	Semi-passive wireless strain gauge sensor to handle fast time-varying phenomena.	Laboratory
[39]	Semi-passive HF RFID tag compatible with ISO/IEC 14443 Type A. Much longer recognition distance than passive tags and can work normally when the magnetic field is 0.3 A/m. Power consumption is as low as 129.6 μW.	Laboratory
[40]	Semi-passive UHF RFID tag with low-power PMU. Manufactured in 0.18μm standard CMOS technology with −26 dBm RF input power.	Laboratory
[41]	Semi-passive UHF sensor tag compatible with ISO 18000-6C. Sensitivity and standby current are −23.7 dBm and 150 nA.	Laboratory
[42]	A passive secure UHF RFID tag and read performance degrades rapidly as conductivity varies to less than 0.5 S/m.	Laboratory
[43]	Wireless strain sensing based on passive UHF RFID tags with high EM isolation between the tags.	Laboratory
[44]	UHF RFID sensing system software-defined radios-based RFID readers.	Laboratory
[45]	RFID sensor antenna with a bandwidth from 778 to 984 MHz and a uniform magnetic field distribution in a large reading region.	Laboratory
[46]	900 MHz is the optimal frequency resulting in a readout distance of up to 7.5 m.	Laboratory
[47]	9 dBiC RFID sensor antenna with optimal detection ranges of 0.57–3.5 m.	Both in the field and laboratory
[48]	UHF RFID sensor and reading distance of RFID tag reduces with the increase in test temperature.	Laboratory
[49]	Contactless RFID system formed by chipless tags based on the magneto-inductive wave delay lines.	Laboratory
[50]	Passive RFID sensors and 900 MHz RFID readers with a link budget of about 100 dB.	Laboratory
[51]	High data capacity chipless RFID sensing system.	Laboratory
[52]	Chipless RFID systems based on near-field coupling between the tag and the reader with sequential bit reading.	Laboratory
[53]	Design and analysis of four conventional and printed monopole and dipole antennas as chipless RFID strain sensors.	Laboratory
[54]	10-bit RFID tag which operates over the frequency range 2.4–3.4 GHz.	Laboratory
[55]	Strain sensor using millimeter wave chipless RFID tag.	Laboratory
[56]	Dual-mode patch antenna sensor fabricated on a substrate material with a steady dielectric constant. Passive patch antenna sensor with good reliability under temperature fluctuations.	Both in the field and laboratory
[57]	Chip-based RFID sensors with two resonances—the resonance of the patch at 3.4 GHz, and the resonance of the loop at 2.9 GHz.	Laboratory
[58]	Operating in the 860 MHz–960 MHz frequency range. Multi-access capability with access rates greater than 1600 tags per second and read speeds at 100 Kbits/s.	Laboratory
[59]	RFID strain sensor with spectral bandwidths of 40 Hz and 26.5 Hz, which has a 30 mHz natural frequency determination error.	Laboratory
[60]	Wireless surface acoustic wave RFID Sensor tolerant for high temperatures, which can measure the strain/stress and temperature on engine blades.	Laboratory
[61]	Reusable passive wireless RFID strain sensor. The tag RL at resonance (878 MHz) is 21.18 dB.	Laboratory
[62]	A phased array using RFID reader antennas to achieve 30 feet (10 m) read range.	Laboratory
[63]	Virtual reader which can closely emulate physical readers by read fail ratio of approximately 10%.	Laboratory

## Data Availability

All data included in this study are available upon request by contact with the corresponding author.
